# Attenuated *Leishmania* induce pro-inflammatory mediators and influence leishmanicidal activity by p38 MAPK dependent phagosome maturation in *Leishmania donovani* co-infected macrophages

**DOI:** 10.1038/srep22335

**Published:** 2016-03-01

**Authors:** Somenath Banerjee, Dipayan Bose, Nabanita Chatterjee, Subhadip Das, Sreeparna Chakraborty, Tanya Das, Krishna Das Saha

**Affiliations:** 1 Cancer Biology and Inflammatory Disorder Division, CSIR-Indian Institute of Chemical Biology, 4 Raja S. C. Mullick Road, Kolkata-700032, India; 2 Department of Molecular Medicine, Bose Institute, Centenary Campus, P 1/12, C. I. T. Road, Scheme– VIIM, Kolkata – 700054, West Bengal, India

## Abstract

Promastigote form of *Leishmania,* an intracellular pathogen, delays phagosome maturation and resides inside macrophages. But till date limited study has been done to manipulate the phagosomal machinery of macrophages to restrict *Leishmania* growth. Attenuated *Leishmania* strain exposed RAW 264.7 cells showed a respiratory burst and enhanced production of pro-inflammatory mediators. The augmentation of pro-inflammatory activity is mostly attributed to p38 MAPK and p44/42 MAPK. In our study, these activated macrophages are found to induce phagosome maturation when infected with pathogenic *Leishmania donovani*. Increased co-localization of carboxyfluorescein succinimidyl ester labeled pathogenic *L. donovani* with Lysosome was found. Moreover, increased co-localization was observed between pathogenic *L. donovani* and *l*ate phagosomal markers *viz.* Rab7, Lysosomal Associated Membrane Protein 1, Cathepsin D, Rab9, and V-ATPase which indicate phagosome maturation. It was also observed that inhibition of V-type ATPase caused significant hindrance in attenuated *Leishmania* induced phagosome maturation. Finally, it was confirmed that p38 MAPK is the key player in acidification and maturation of phagosome in attenuated *Leishmania* strain pre-exposed macrophages. To our knowledge, this study for the first time reported an approach to induce phagosome maturation in *L. donovani* infected macrophages which could potentiate short-term prophylactic response in future.

The protozoan parasite *Leishmania* infects phagocytes causing a spectrum of diseases from less severe cutaneous leishmaniasis to lethal visceral leishmaniasis (VL). Pathogenic *L. donovani* (PLD; AG83/MHOM/1983), a protozoan parasite, causes VL or Kala-azar, which is prevalent in tropical and temperate regions^1^. Leishmaniasis is endemic in 88 countries with an estimated yearly incidence of 1–1.5 million cases of cutaneous leishmaniasis and 500,000 cases of VL, of which 70,000 people die every year^2^,^3^. Treatment options for leishmaniasis include sodium antimony gluconate (SAG or SbV), Miltefosine, Pentamidine, and Amphotericin B which are drugs with serious limitations due to toxicity or resistance^4^. Development of vaccines to thwart leishmaniasis has been an objective for a century now. First efforts at vaccination, termed leishmanization have been largely terminated due to a variety of reasons. The first-generation vaccines based on killed parasites have substituted leishmanization, but evidence from studies in animal models has been found inadequate in field studies^5^. Second generation vaccines employing genetically modified parasites have not proved their competence so far^6^.

*Leishmania* exists in two forms namely promastigotes, which is found in the sand fly and gets transferred to human or other mammals during a blood meal, and amastigotes. Promastigotes differentiate into amastigotes which take almost 120 h to complete^7^, when they resist the microbicidal machinery of the host cell especially by manipulating phago-lysosome formation. Uptake of metacyclic promastigotes is done by specialized phagocytes macrophages (MΦs), while neutrophils serve as the Trojan horse for *Leishmania* to get entry into MΦs^8^. But phagocytosis does not necessarily mean the elimination of microbes; rather a complex array of events leading to maturation of phagosome needs to be proficient. But *Leishmania* maneuver the phago-lysosomal machinery of MΦs to ensure their survival by inhibiting phagosomal maturation, disrupting phagosome micro-domains, excluding NADPH oxidase (NOX) and proton-ATPase from phagosomal membrane, accumulating peri-phagosomal actin, reducing recruitment of the late phagosome marker Rab7 and delaying recruitment of LAMP1 probably by impairment in Rab7 recruitment as reviewed by Moradin *et al.*^9^. Other strategies to survive inside MΦs include modulation of Nitric Oxide (NO) generation, NOX activation, and Reactive Oxygen Species (ROS) production, hampering expression of inducible Nitric Oxide Synthase (iNOS), IL-12 production, and NF-κB and AP-1 activation. Resolution of infection depends not only in eluding the effector arms but also inducing anti-inflammatory cytokines like IL-10, TGF-β, and IL-4 as reviewed by Bhardwaj *et al.*^10^. To create this favorable niche for *Leishmania*, Mitogen Activated Protein Kinases (MAPK) plays a critical role^11^. It has been reported that activation of the p-44/42 MAPK leads to induction of IL-10, while downregulation of p38 MAPK is responsible for abrogated IL-12 production^12^,^13^.

While raising a successful adaptive immune response against *Leishmania* is difficult, triggering an innate immune modulation to uproot *Leishmania* from the MΦ in the early hour of infection by means of accomplishing phagosome maturation has never been tried to date. In contrast to PLD, its counterpart Attenuated *Leishmania* Strain (ALS; UR6/MHOM/1978) has been reported to boost Superoxide burst and TNF-α generation in peritoneal MΦs^14^. LPG defective mutants of *Leishmania* are also reported to fuse with endosome extensively^15^. There are reports on *Leishmania major* which raise non-specific antimicrobial defense, including up-regulation of pro-inflammatory mediators^16^. So, studies on post infection of PLD in macrophages, already infected with ALS, could provide an interesting read out on innate immunity against *Leishmania* infection.

Co-infection studies have been reported in the field of pathogenesis of infectious diseases and findings on some of them were encouraging. *Coxiella burnetti*, the agent of Q fever, is another intracellular pathogen that replicates in large spacious phago-lysosome^17^ which is acidic^18^ and fusogenic^19^ in nature and has been successfully used in experiments to reduce survival and growth of mycobacteria by co-infection^20^. Co-infection of *Leishmania* with *Plasmodium* also shows better Th1 response and lower *Plasmodium* survival^21^. In another case of *C. burnetti* infection, there was less invasion of trypomastigotes, while increasing the vacuolar pH using bafilomycin caused greater invasion, thus suggesting that acidification of *C. burnetti* vacuoles plays the trick^22^. *Schistosoma mansoni* and *L. major* co-infection on other hand showed protection against *S. mansoni* but pathology due to *L. major* infection increased^23^. Hence in our present study LPG-deficient strain ALS was used to modify the MΦ response towards PLD.

In this study, we first observed the response of ALS mono-infected MΦs and then of MΦs co-infected with ALS and PLD. ALS pre-exposed MΦs showed altered response upon infection with PLD. MΦs that is pre-exposed to ALS have profound production of pro-inflammatory molecules including NO and IL-12, which are important players in the battle against *Leishmania*. ALS is reported to be deficient of LPG^24^ which is important because LPG defective mutant show extensive phagosome maturation^15^ and we observed indeed that ALS pre-treated MΦs showed increased phago-lysosome fusion when infected with PLD. In depth study confirm p38 MAPK as the key player in MΦs activation as well as phagosomal maturation event in ALS pre-exposed MΦs.

## Results

### Augmentation of superoxide generation in ALS treated MΦs

**Figure 1:**
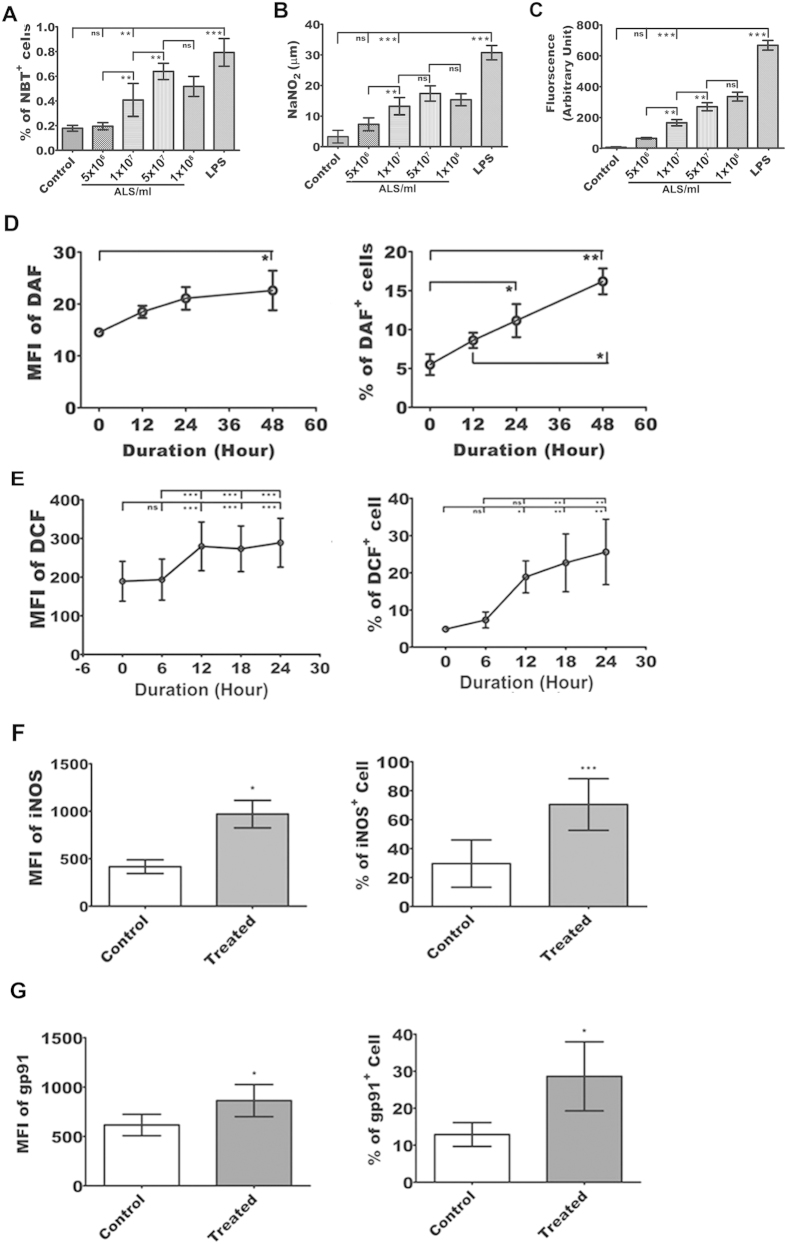
ALS exposure produces respiratory burst in RAW 264.7 cells. (**A–C**) 1 × 10^6^ RAW 264.7 cells/ml was incubated with or without 5 × 10^6^, 1 × 10^7^, 5 × 10^7^, and 1 × 10^8^ ALS or LPS for 4 h, washed, fresh media was added, and assayed after 12 h. Dose kinetics of the levels of NBT (**A**), NO (**B**) and ROS (**C**) were measured. (**D,E**) 1 × 10^6^ RAW 264.7 cells/ml was incubated with or without 1 × 10^7^ ALS for 4 h and processed as before. Flowcytometric analysis of NO (**D**) and ROS (**E**) production was evaluated in a time dependent manner using DAF-FM and DCF respectively. (**F,G**) In ALS treated MΦs iNOS (**F**) and gp91 (**G**) levels were observed. Change in MFI and percent positive cells was represented graphically for each of the above experiments. Data are representative as the mean ± SD and are the cumulative results of three independent experiments *p < 0.05, **p < 0.01, ***p < 0.001.

It is well established that *Leishmania* abolishes the superoxide generation in MΦs for its survival. Studies showed that augmentation of superoxide generation inhibited *Leishmania* growth. Therefore we sought to monitor superoxide generation in ALS treated MΦs. Raw 264.7 cells were incubated with different concentrations of ALS, viz. 5 × 10^6^, 1 × 10^7^, 5 × 10^7^ and 1 × 10^8^ cells per ml and then p-nitro blue tetrazolium (NBT) reduction assay was performed. The optimum increment in superoxide generation (P < 0.05) was observed (Fig. 1A) in MΦs primed with 1 × 10^7^ ALS.

To confirm the finding, we further checked the ROS and NO production in ALS treated MΦs. The sharp rise in ROS and NO production was observed (Fig. 1B,C) in MΦs treated with 1 × 10^7^ ALS, (P < 0.05). In a time dependent study, increased NO generation was observed at 24 h (Fig. 1D), whereas a steady state gradual increase in ROS generation was noticed up to 24 h (Fig. 1E) (P < 0.05). Therefore, a promising microbicidal activity was anticipated due to the augmentation of superoxide generation, which is an essential attribute for combating pathogens.

### Increased expression of iNOS, and gp91 phox in ALS primed MΦs

Among the three types of Nitric oxide synthases, iNOS is responsible for anti-microbial NO production while gp91^phox^, a subunit of NADPH oxidases, is one of the key components of microbicidal oxidases in phagocytes^25^. Our flowcytometric data showed augmented expressions of iNOS (Fig. 1F) and gp91^phox^ (Fig. 1G) in ALS primed MΦs (P < 0.05). These further validate the capability of ALS to induce respiratory burst in MΦs.

### ALS treated MΦs showed enhanced phagocytic activity

**Figure 2:**
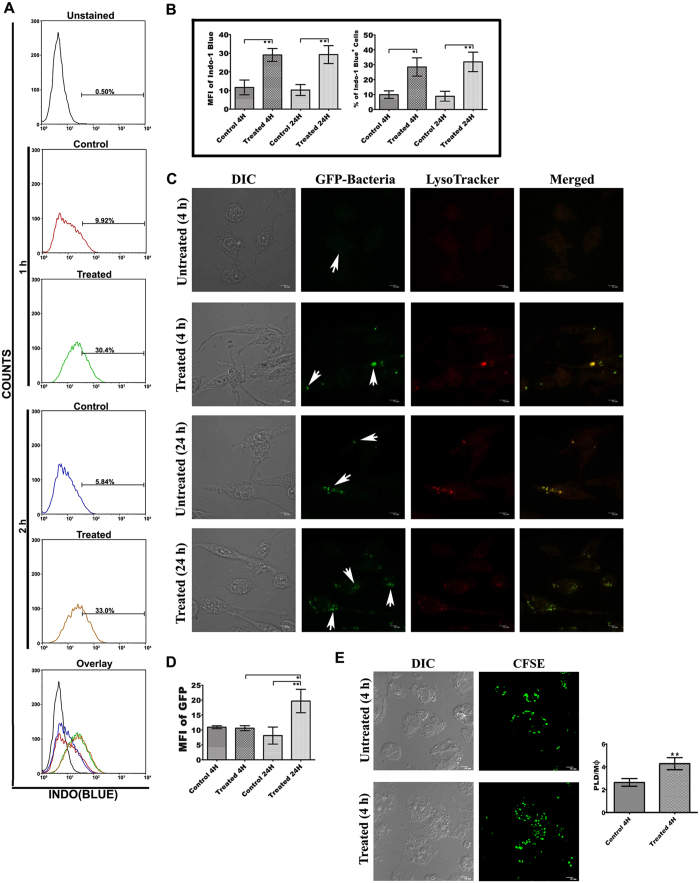
Effect of ALS pre-treatment on phagocytosis. (**A**) RAW 264.7 cells were treated with or without 1 × 10^7^ ALS for 4 h, then washed and incubated with fresh media containing fluorescence latex beads for 1 and 2 h. Phagocytosis was studied by measuring the fluorescence intensity of engulfed latex bead. (**B**) Graphical representation depicted the latex beads containing percent positive cells and mean fluorescence intensity of engulfed latex beads in ALS treated/untreated cells. (**C**) ALS treated MΦs were incubated with GFP-expressing bacteria for 4 and 24 h and phagocytosis was studied under confocal microscope. (**D**) Change of mean fluorescence intensity due to phagocytosis of GFP-expressing bacteria was shown in above mentioned condition. (**E**) Cells were treated with ALS as before. After 4 h cells were again infected with CFSE labeled AG83 for another 4 h and uptake of CFSE labeled AG83 was observed under confocal microscope. Data are representative as the mean ± SD and are the cumulative results of three independent experiments *p < 0.05, **p < 0.01, ***p < 0.001. All the confocal microscopic data were analyzed by using ImageJ software.

ALS treated MΦs showed enhanced phagocytosis of latex bead. Engulfment was found to increase with time (Fig. 2A,B) (P < 0.05). Our Confocal microscopic data also showed increased phagocytosis of GFP-expressing *E. coli* DH5 (α) in ALS pre-treated MΦs (Fig. 2C,D) (P < 0.05). Similarly, treated MΦs showed enhanced uptake of PLD as compared to untreated ones (Fig. 2E). While it was reported that increment of phagocytic activity was not a critical hallmark of classical activation of MΦs, the present observation is important because PLD infection restricts the phagocytic capacity of MΦs^26^.

### Pre-exposure to ALS stimulates TNF-α, IL-1β, and IL-12 production in MΦs

**Figure 3:**
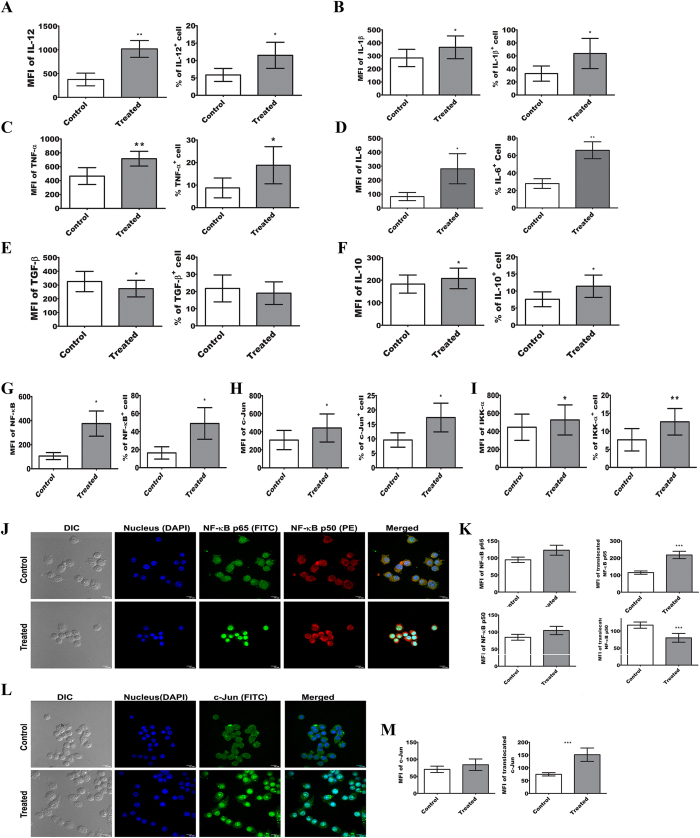
Up-regulation of pro-inflammatory mediators in MΦs upon treatment with ALS. (**A–F**) IL-12 (**A**), IL-1β (**B**), TNF-α (C), IL-6 (**D**), TGF-β (**E**), and IL-10 (**F**) levels were observed by flowcytometry. RAW 264.7 cells were infected with 1 × 10^7^ ALS for 4 h, then washed; fresh media was added and incubated for 12 h before processing for flowcytometry. (**G–I**) RAW 264.7 cells were incubated with 1 × 10^7^ ALS for 4 h, washed, fresh media was added, incubated another for 4 h and processed for flowcytometric study of NF-κB (**G**), c-JUN (**I**), and IKK-α (**J**). MFI and percent positive cells were represented graphically for each of the above experiments. (**J,K**) RAW cells were primed with 1 × 10^7^ ALS for 4 h, washed, fresh media were added and again incubated for 4 h, and nuclear translocation of NF-κB p65, NF-κB p50 (**J**) was observed. Expression of NF-κB p65 (left lower panel) and p50 in the cell (left upper panel) and in the nucleus (right panel) was graphically shown (**K**). (**L,M**) RAW cells were primed with 1 × 10^7^ ALS for 4 h, washed, fresh media were added and again incubated for 4 h, and nuclear translocation of c-JUN was observed. (**L**). Expression of c-JUN in whole macrophage (left panel) and nuclei (right panel) was graphically represented. Data are representative as the mean ± SD and are the cumulative results of three independent experiments *p < 0.05, **p < 0.01, ***p < 0.001.

In order to determine the effect of ALS, in the production of pro-inflammatory cytokines in MΦs, expression of IL-12 (Fig. 3A), IL-1β (Fig. 3B), TNF-α (Fig. 3C), ΙL−6 (Fig. 3D), ΤGF−β (Fig. 3E), and IL-10 (Fig. 3F) were checked. While Th1 type cytokines *viz.* IL-12, IL-1β, TNF-α, and IL-6 showed a significant rise (Fig. 3A–D) in ALS treated MΦs (P < 0.05), expression of Th2 type cytokine i.e., TGF-β was reduced (Fig. 3E) (P < 0.05), though another anti-inflammatory cytokine IL-10 expression was increased (Fig. 3F) (P < 0.05). Therefore, it can be speculated that enhanced pro-inflammatory cytokines production may alter the intracellular microenvironment of macrophage towards leishmanicidal type.

### Elevation of expression and nuclear translocation of NF-κB and c-JUN in ALS treated MΦs

We next sought to determine the probable factors working behind the production of microbicidal mediators in ALS treated MΦs and also to monitor the expression of transcription factors that have been reported to associate with it. We observed the expressions as well as nuclear translocation of NF-κB and c-JUN, which are the key transcription factors associated with the production of pro-inflammatory mediators. Our Flowcytometric analysis revealed significant up-regulation (P < 0.05) of NF-κB p65 (Fig. 3G), c-Jun (Fig. 3H), and IKK-α (Fig. 3I) in ALS primed MΦs. Nuclear accumulation of NF-κB p65 and c-Jun was also Increased (Fig. 3J–M) in MΦs pre-exposed to ALS for 4 h (P < 0.05). Simultaneously, there was a decreased translocation of NF-κB p50 (Fig. 3J,K) (P < 0.05), which further ensured the functional activity of NF-κB^27^, the key factor regulating expression of TNF-α, IL-12 and iNOS^28^.

### Activation of MAPK in ALS treated MΦs

**Figure 4:**
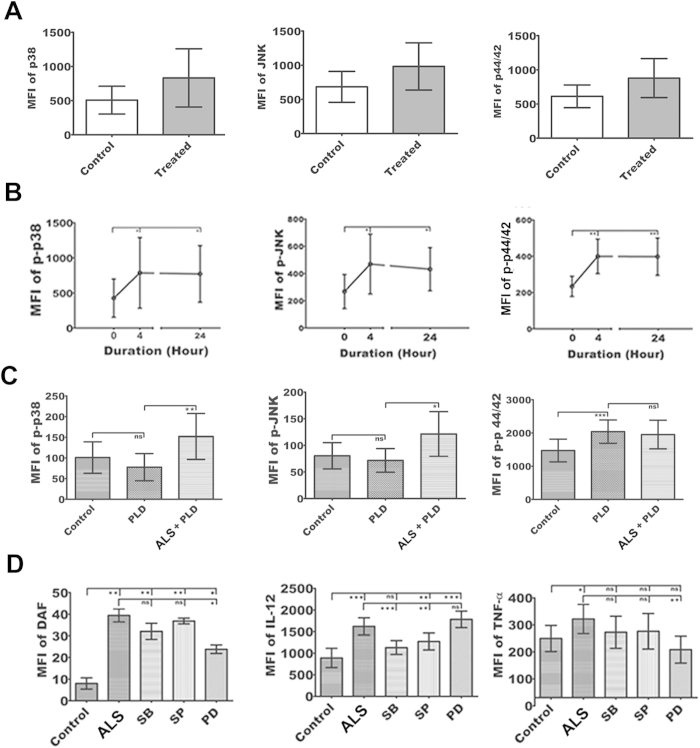
MAPK Signaling events in MΦs primed with ALS. (**A**) RAW 264.7 cells were primed without or with 1 × 10^7^ ALS for 4 h, then washed; fresh media was added and incubated for another 20 h. Native p38, JNK, and p44/42 MAPK levels were shown graphically. (**B**) RAW 264.7 cells were primed without or with 1 × 10^7^ ALS for 4 h, p-p38, p-JNK, and p-p44/42 MAPK levels were observed immediately and after 20 h of further incubation in fresh media. Percent positive cells and MFI values were shown graphically. (**C**) RAW 264.7 cells were primed with 1 × 10^7^ ALS for 4 h, then washed; and fresh media was added. After 12 h these MΦs were infected with PLD for 4 h. Graphical representation depicted percent positive cells and MFI values of p-p38, p-JNK, and p-p44/42 MAPK. (**D**) MΦs were infected for 4 h with 1 × 10^7^ ALS, then washed, and in fresh media SB203580, SP600125, and PD098059 were added. After 4hproduction of NO, IL-12 and TNF-α was measured. Data are representative as the mean ± SD and are the cumulative results of three independent experiments *p < 0.05, **p < 0.01, ***p < 0.001.

Since MAPKs constitute the central hub for a wide variety of cellular function, including the effect on different cytokine production in MΦs, we decided to explore the upstream signaling event involved in ALS mediated MΦ activation. Several reports suggested that *Leishmania* infection alters the MAPK signaling^11^ and low intensity signal from CD40 signalosome cause significant up-regulation of p44/42 MAPK that leads to augmentation of IL-10 production in *Leishmania* infected MΦs^12^. In our study, although the expressions of p38 MAPK, p44/42 MAPK and JNK were unaltered by ALS treatment (Fig. 4A), p-p38 MAPK, p-JNK and p-p44/42 MAPK level were significantly up-regulated and remained elevated till 24 h of ALS exposure (Fig. 4B) (P < 0.05).

Interestingly, when these ALS pre-treated MΦs were infected with PLD, no suppression of p38 MAPK and JNK (Fig. 4C) was observed (P < 0.05). On the other hand, in ALS pre-exposed MΦs, elevated p-p44/42 MAPK level was normalized after infection with PLD (Fig. 4C) (P < 0.05).

For further confirmation, we used inhibitors of p38 MAPK, JNK, and p44/42 MAPK. Result showed that p44/42 MAPK is an important regulator of NO production in ALS treated MΦs, while p38 MAPK is responsible for IL-12 production (P < 0.05) (Fig. 4D). However, the increment of TNF-α production after treatment with ALS was under tripartite regulation involving all the three MAPKs (Fig. 4D) (P < 0.05). Altogether this indicated that p38 and p44/42 MAPK were essential for ALS mediated MΦ activation^13^,^29^.

### Phagosome maturation conferring leishmanicidal attribute to ALS pre-treated MΦs

**Figure 5:**
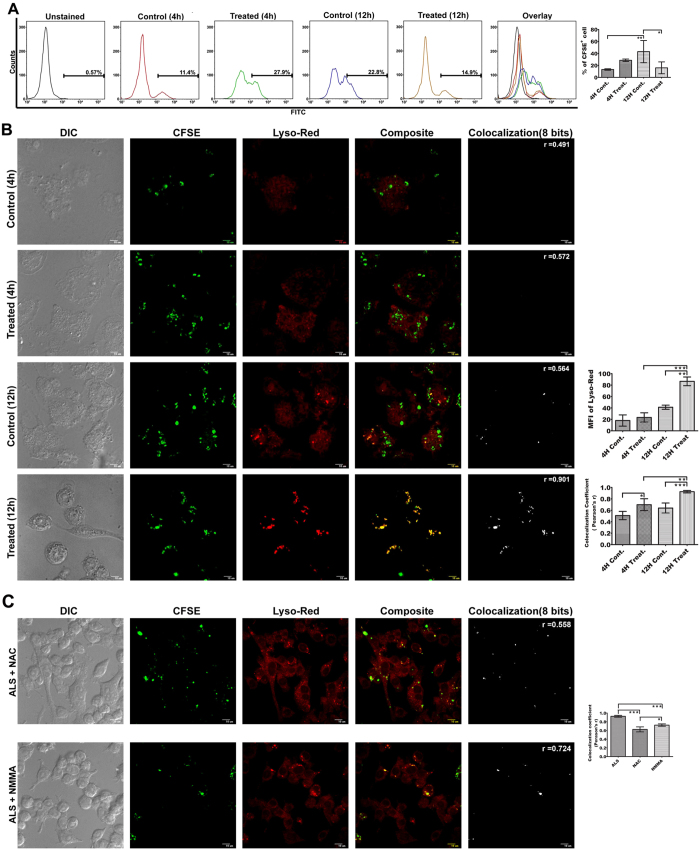
Phagosome maturation in PLD loaded ALS primed MΦs. MΦs were primed with 1 × 10^7^ ALS for 4 h, washed, after 12 h CFSE labeled PLD was added, and incubated for further 12 h. (**A**) Viable parasite in ALS untreated/treated MΦs was analyzed by flowcytometry. (**B**) Co-localization of CFSE labeled PLD with Lysotracker red which specifies the acidic Lysosomal compartments, was observed under confocal microscope (left panel). Co-localization index (Pearsons’ r) were graphically represented (right panel). (**C**) MΦs were infected with 1 × 10^7^ ALS for 4 h, washed, and then treated with NAC and NMMA. After 4 h cells were washed and incubated for further 8 h in fresh media. These MΦs were then infected with CFSE labeled PLD for another 4 h, washed, fresh media was added. After 8 h post infection Co-localization of CFSE labeled PLD with Lysotracker red was observed (left panel). Co-localization index (Pearsons’ r) was graphically represented (right panel). Data are representative as the mean ± SD and are the cumulative results of three independent experiments *p < 0.05, **p < 0.01, ***p < 0.001.

We next sought to determine whether ALS pre-treatment has any effect on parasite viability within those activated MΦs. For this ALS primed MΦs were infected with CFSE labeled PLD and viability were observed by flowcytometry. In comparison to untreated MΦs, ALS primed MΦs were loaded with a higher number (Fig. 5A) of viable PLDs after 4 h of infection (P < 0.05). Noticeably, at 12 h there was a decrease in fluorescence intensity of CFSE labeled PLD, which indicates enhanced parasite killing (P < 0.05) as compared to untreated MΦs (Fig. 5A).

However, since the intracellular pathogen *Leishmania* resides in the phagosome, it significantly hinders fusion of the phagosome with lysosome^15^. So, our next effort was to delineate the extent of phagosome-lysosome fusion in ALS treated MΦs. We found higher co-localization (P < 0.05) between CFSE labeled PLD and lysosome (Fig. 5B) which indicates increased phago-lysosome biogenesis, whereas Fusion of PLD and lysosome remained unaltered in untreated MΦs throughout the time point. Involvement of ROS and NO in inducing phagosome maturation is well established^30^. Treatment of NAC and NMMA in ALS pre-treated MΦs were shown to inhibit ROS and NO generation respectively (Supplementary Fig. S1). In our system inhibition of ROS generation was found to prevent phagosome maturation (Fig. 5C), rather than does by inhibition of NO generation (P < 0.05). This is particularly striking because it was reported that NO plays a major role in overcoming the blockade of phagosome maturation by rupturing the peri-phagosomal actin formed in the periphery of *Leishmania* loaded phagosomes^30^.

**Figure 6:**
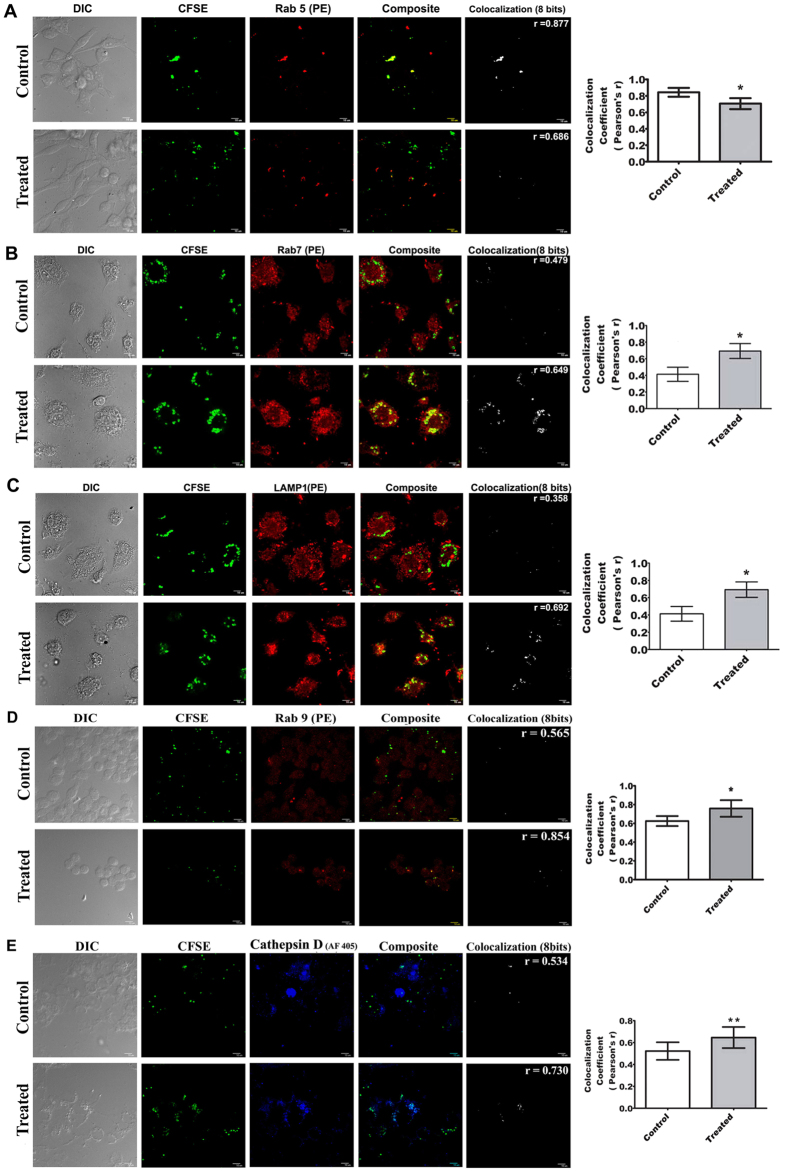
Co-localization of various phagosomal markers with CFSE labeled PLD. RAW 264.7 cells were primed with 1 × 10^7^ ALS for 4 h, washed and fresh media was added. After 12 h CFSE labeled PLD was added and incubated for further 12 h. Co-localization of RAB5 (**A**), RAB7 (**B**), LAMP-1 (**C**), RAB9 (**D**), and Cathepsin D (**E**) with CFSE labeled PLD was studied in MΦs. Co-localization index (Pearsons’ r) were graphically represented (right panel). Data are representative as the mean ± SD and are the cumulative results of three independent experiments *p < 0.05, **p < 0.01, ***p < 0.001.

Rab5 is a well-known early endosomal marker^31^. We found that Rab5 increasingly dissociated from CFSE labeled parasites (Fig. 6A) (P < 0.05), indicating a loss of early markers of phagosome. It is established that Rab7 and Lamp1 are the two key markers of late phagosomal activity^32^,^33^. Though previous report suggested that Rab7 recruitment is delayed in *Leishmania* loaded phagosome^34^, in our study both Rab7 and LAMP1 molecules showed higher co-localization with CFSE labeled PLD after 12 h of infection in ALS primed MΦs (Fig. 6B,C) (P < 0.05). Our previous results showed an up-regulation of IL-12, which was reported to induce p38 dependent Rab7 expression in MΦs^35^ and that may be the reason for greater Rab7 accumulation in PLD loaded phagosomes of ALS pre-treated MΦs. Mature Cathepsin D is also a known non-oxidative marker for late phagosome^36^ and Rab9 is recognized for its function related to recycling of the endosome that has already undergone phago-lysosomal maturation process; thus these molecules also serve as markers of late phagosomal activity^37^. We found that there is increased co-localization of CFSE labeled parasites with both these molecules (Fig. 6D,E) (P < 0.05), which further validated enhanced maturation of parasite loaded phagosomes that serve as the prerequisite for killing an intracellular pathogen. Another key marker of maturing phagosome is V-ATPase, which serves to acidify the lumen of the endosome^38^. In our study we observed increased co-localization of V-ATPase and CFSE labeled PLD in ALS pre-treated MΦs as compared to their untreated counterparts (Supplementary Fig. S2). From these data, the involvement of both V-ATPase and cathepsin D in phagosome maturation was speculated in ALS pre-treated MΦs.

**Figure 7:**
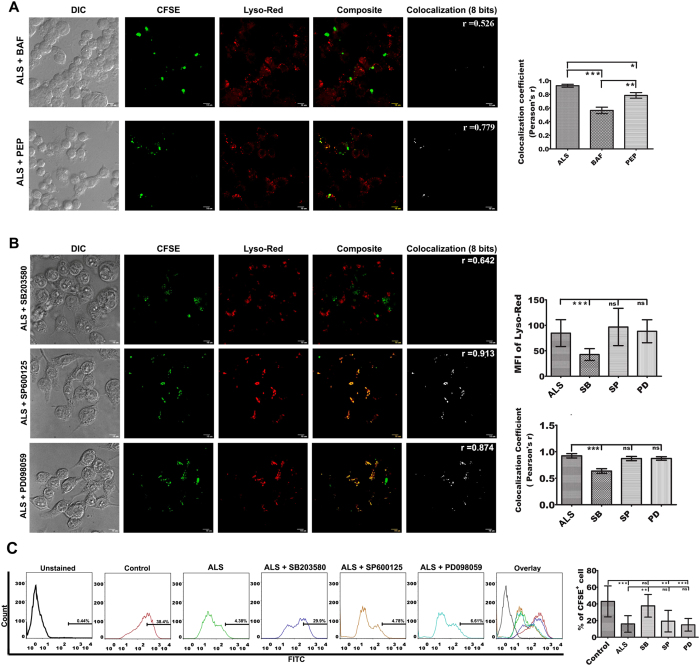
Involvement of p38 MAPK in phagosome maturation. (**A**) MΦs were infected with ALS for 4 h, washed, and incubated for further 8 h in fresh media. These MΦs were then infected with CFSE labeled PLD for 4 h and then treated with bafilomycin A1 and pepstatin A for 4 h and then washed, fresh media was added. After 12 h post infection, co-localization of LysoRed and CFSE labeled PLD was observed under a microscope (left panel). Co-localization index (Pearsons’ r) were graphically represented (right panel). (**B**) RAW 264.7 cells were infected with ALS for 4 h, washed, and fresh media was added and incubated for additional 12 h. These MΦs were then infected with CFSE labeled PLD for 4 h and then treated with SB203580, SP600125, and PD098059 for another 4 h. Cells were then washed and incubated for further 8 h in fresh media. Co-localization of LysoRed and CFSE labeled PLD was observed under microscope (left panel). Co-localization index (Pearsons’ r) were graphically represented (right panel).(**C**) MΦs were primed with ALS, infected with PLD and treated with MAPK inhibitors as stated above. Viable parasite load was determined by flowcytometry (left panel). Percent positive cells were represented graphically (right panel). Data are representative as the mean ± SD and are the cumulative results of three independent experiments *p < 0.05, **p < 0.01, ***p < 0.001.

Both V-ATPase and cathepsin D were reported to be excluded from the phagosome by *Leishmania* through manipulation of synaptotagmin-V dependent procedure^39^. In our study we observed that inhibition of only cathepsin D using pepstatin A did show some degree of inhibition in phago-lysosome fusion in ALS pre-treated MΦs, whereas inhibition of V-ATPase by bafilomycin A1 significantly hampered the process (Fig. 7A) (P < 0.05). This indicated that induction of acidification of phagosomes loaded with PLD is much critical for phago-lysosomal biogenesis in ALS pre-treated MΦs.

### p38 MAPK regulates acidification and maturation of PLD loaded phagosome

Interestingly, p38MAPK which induced pro-inflammatory molecules in ALS pre-treated MΦs is also one of the key molecules that participate in phagosomal maturation^40^,^41^ and attenuate *Leishmania* growth within the host MΦs^40^. In order to ascertain the role of p38 MAPK in phago-lysosome fusion in our system, we used inhibitors of MAPK. While inhibition of JNK and p44/42 MAPK could not halt the fusion of *Leishmania* loaded phagosomes with lysosome, use of a p38 MAPK inhibitor almost fully hindered the phagosome maturation and acidification process (Fig. 7B) (P < 0.05). Moreover, inhibition of p38MAPK also significantly decreased the leishmanicidal activity (P < 0.05) of ALS pre-exposed MΦs (Fig. 7C). Hence, p38 MAPK can be considered as the key signaling molecule, which induces leishmanicidal activity via modulation of phago-lysosomal biogenesis in ALS pre-treated MΦs.

## Discussion

*Leishmania* has an extraordinary propensity to survive in the hostile environment of the host and suppress the host defense system^9^,^30^. But the attenuated version of this parasite was reported to boost TNF-α and NO generation in murine MΦs^14^. In line with this, our present study has described Th1 cytokine bias along with respiratory burst in ALS pre-exposed MΦs. For the first time, this study demonstrates that ALS pre-exposure surprisingly induce phagosome maturation in PLD infected MΦs. Augmentation of both NO and ROS have encouraged us to speculate about its leishmanicidal activity because it is well known that NO and ROS combine with peroxynitrite to produce the strongest microbicidal weapon in defense against *Leishmania*^42^. Moreover, it was also observed that the already internalized PLD failed to limit the uptake of further PLD in ALS treated MΦs which is important for overall fitness for the survival of the parasites.

In harmony with superoxide generation, there was clear enrichment of IL-12, IL-1β, and TNF-α in MΦs after ALS treatment. One school of thought emphasizes that the promotion of Th1 over Th2 response in *Leishmania* infection obstructs disease progression^43^. Thus we hypothesize that ALS induced Th1 bias might inhibit survival of *Leishmania* in MΦs. On the way to explore the molecular events which dictate the skewing of MΦ function to Th1 bias, we observed enhanced expression and nuclear accumulation of NF-κBp65 and c-jun in ALS treated MΦs whereas NF-κB and AP-1 (a heterodimer of fos and jun or homodimer of jun) activity is hindered in *Leishmania* infected MΦs^29^. In addition to this, there was decreased nuclear translocation of p50 which might be relevant in this context as because trans-activation capability of p65 is reduced during co-expression with p50, which has the competitive DNA binding activity with p65^27^.

Establishment of infection occurs due to the differential production of the counter regulatory cytokines IL-10 and IL-12 which are controlled by p44/42 MAPK and p38 MAPK respectively^12^. Though both these MAPK were up-regulated in ALS treated MΦs, the increase in IL-12 production was much higher than that of IL-10. Surprisingly, the up-regulation of p-p44/42 MAPK which is responsible for IL-10 production was significantly inhibited when ALS pre-treated MΦs were further challenged with PLD. On the other hand, the p-p38 MAPK, which is responsible for IL-12 production remained up-regulated in ALS pre-treated MΦs even after PLD infection.

Heightened phosphorylation of p38 which is also effective in attenuating *Leishmania* virulence^40^ substantiates the speculation of enhanced phagosomal maturation^40^,^41^ in these macrophages. Moreover, Pro-inflammatory mediators have been reported to trigger phagosomal maturation process^30^,^44^; whereas anti-inflammatory cytokines tend to dampen the process^45^. Based on these evidence we were first keen to find if these activated MΦs are equipped with leishmanicidal activity. We ascertained that infection of viable PLD in ALS pre-exposed MΦs leads to a collapse in a number of viable parasites with time which points towards enhanced leishmanicidal activity. Next, we want to monitor the phago-lysosome fusion which might be an interesting target to reduce the establishment of infection^46^. We observed significant interaction of CFSE labeled PLD with lysosome, and increased phagosomal acidification in ALS treated MΦs. It is now well established that phagosome maturation continues with a loss of early endosome marker *viz.* Rab5 and subsequent acquisition of late endocytic marker *viz.* Rab7^31^,^47^. MΦs with active mutant of Rab5 expression are shown to be not only 5–10% less efficient in killing *Leishmania* but also to restrict complete fusion of the phagosome with endosome^48^. In our study, loss of Rab5 positive phagosome containing PLD indicated the beginning of phagosome maturation in ALS pre-treated MΦs. Simultaneously, Rab7 and LAMP1 showed increased co-localization with CFSE labeled PLD in ALS pre-exposed MΦs which further emphasized phagosome maturation. In ALS treated MΦs, Rab9 showed higher co-localization with CFSE labeled parasites, which is important because Rab9 is also involved in lysosomal trafficking of ingested particles^49^.

Acidification of phagosome is the most important readout because cathepsins become active only when phagosome attains sufficiently low pH^50^. V-type ATPases, which are central to the fall of pH, are kept aside from the *Leishmania* containing phagosome for up to 24 h^38^,^39^. Phagosome containing amastigotes that are devoid of LPG acquire several markers of late phagosome or phago-lysosome^39^,^51^. But unlike amastigotes, promastigotes cannot withstand at low pH and thus phagosome maturation is delayed which offers a timeframe during which they can differentiate to amastigotes. It is known that PLD containing phagosome has a pH of 5.5 which may rise up to 5.8 and exacerbate the spread of infection^52^. This is a much higher pH that makes PLD containing phagosomes incapable of acquiring active cathepsin D^51^. It has been reported that both V-type ATPase and cathepsin D were excluded from PLD loaded phagosome^39^,^51^. But in our study, CFSE labeled parasites were shown to co-localize with cathepsin D in ALS pre-treated MΦs, a non-oxidative marker of phagosome maturation. Our results also provide the insight that probably the fall of pH is the key regulating factor for promastigote growth inhibition in ALS pre-treated MΦs as because Inhibition of V-ATPase by bafilomycin A1 was found to have a major impact on the phagosome maturation as compared to pepstatin A, an inhibitor of cathepsin D. So, it may be postulated that V-ATPase is more important than cathepsin D in phagosome maturation process of *Leishmania* loaded ALS pre-treated MΦs.

It is well established that IL-12 is responsible for up-regulation of Rab7 in a p38 MAPK dependent manner^35^; LAMP-1 expression is also reported to be up-regulated by IL-12 stimulation in NK cells^53^. It has been proved that supplying IL-12 to MΦs infected with *M. tuberculosis* enhances phago-lysosomal biogenesis and acidification of phagosomal lumen^44^. Even p38 MAPK was involved in phosphorylation of Rab5 GDI to enhance endosome maturation^54^. So we hypothesize that IL-12 secreted by ALS pre-treated MΦs may work in an autocrine manner to induce p38 MAPK, which can modulate the phagosomal event. Results of our experiments using MAPK inhibitors confirmed that p38 MAPK was solely responsible for maturation of PLD loaded phagosomes and its subsequent leishmanicidal activity. Whether heightened IL-12 production paves the way for this maturation process is yet to be determined but the role of p38 MAPK is established in this study. So, it will be fascinating to investigate in future whether participation of autocrine IL-12 alone or TLR2/4 axis during phagocytosis of ALS has any role in the induction of p38 MAPK as well as in phagosome maturation.

Though phagocytosis serves as the pre-requisite to eliminate the invading pathogens, the paradox is that it could also serve as a portal of infection and provide unintended benefits to intracellular pathogens. But it is established in our study that pre-exposure of ALS to MΦs shows a promising pro-inflammatory attribute essentially by up-regulating p38 MAPK, which makes PLD infected MΦs capable of driving phagosome maturation. These findings are likely to be beneficial for the short-term prophylactic response during a visit to endemic areas identified for *Leishmania* infection. Moreover, attenuation of phagosome maturation which is a hallmark of intracellular pathogen infection was surmounted by ALS treatment; which might be effective in combat against other intracellular infections.

## Materials and Methods

### Chemicals

Lipopolysaccharide (LPS, 0111:B4), and brefeldin A were purchased from Sigma Chemical Company, St. Louis, Mo, USA and NBT from Amresco, USA. Roswell Park Memorial Institute medium-1640 (RPMI-1640), M-199 medium, fetal bovine serum (FBS), penicillin–streptomycin (PS), Gentamycin, and HEPES were purchased from Gibco BRL, Grand Island, NY, USA. Brain Heart infusion and Luria agar were obtained from HiMedia, Mumbai, India. Tissue culture plastic wares were procured from NUNC (Roskilde, Denmark) and DAPI (4′,6-diamidino-2-phenylindole dihydrochloride), Lysotracker Red and CFSE from Invitrogen, CA. All the primary and secondary antibodies were obtained from Santa Cruz Biotechnology, Santa Cruz, CA or Cell signaling technologies, USA. Assay kits for cytokine measurements were purchased from Thermo Scientific. DAF-2 DA (4,5-diaminofluorescein diacetate) and DCFDA (2′,7′-dichlorofluorescein diacetate) were purchased from Calbiochem, USA.

### Macrophage culture

RAW 264.7 cells were obtained from the American type Culture Collection (ATCC). RAW 264.7 cells were suspended in RPMI-1640 supplemented with 10% heat inactivated FBS, 10 μg/ml of gentamycin, and 100 μg/ml of PS.

### Parasite culture

Attenuated *Leishmania* strain UR-6 (ALS) strain was routinely maintained in Brain Heart Infusion at 22 °C. Pathogenic *Leishmania donovani* AG83 (PLD) promastigotes were cultured at 22 °C in M-199 medium containing 10% FBS and PSN^55^.

### Parasite treatment

1 × 10^5^–1 × 10^8^ cells/ml ALS was used to treat macrophages. In case of infection of macrophages with PLD promastigotes, a ratio of 1:10 (MΦs: parasite) was used. Each time MΦs were incubated with ALS washed, and then either fresh media was added or treated with inhibitors and prepared for respective assays after the requisite time of incubation. To study the phagosome maturation or leishmanicidal activity, these ALS pre-treated MΦs were subjected either to direct PLD infection or initially treated with inhibitors, then washed and further infected with PLD. Then additional incubation was done in fresh media and prepared for experiments. A schematic representation of the infection and treatment protocol was shown in Supplementary Fig. S3.

### Measurement of iNOS, gp91^phox^, intracellular cytokine, NF-κB and c-JUN

For the determination of intracellular cytokines, cells were treated with 10 μg/ml of brefeldin A for 3 h. Then they were washed with PBS, fixed with methanol, perforated by saponin and finally labeled with primary and fluorochrome conjugated secondary antibody.

For iNOS and gp91^phox^ determination cells were washed with PBS, then fixed with methanol, perforated by saponin solution (0.2%) and labeled with primary and respective fluorochrome conjugated antibodies. For measurement of NF-κB and c-JUN, the above procedures were followed except that Triton-x 100 solution (0.5%) was used for perforation.

Cells were then analyzed in BD FACS Verse or BD FACS LSR Fortessa. Total 10,000 events were acquired and analyzed using the trial version of FLOWJO software.

### Nitric Oxide measurement

Nitrite level in the culture supernatant was measured using the Nitric Oxide Colorimetric Assay kit that utilizes Griess reaction^29^. Briefly, 1 × 10^6^ cells were treated with 1 × 10^5^–1 × 10^8^ ALS/ml for 24 h and the supernatants were used to measure NO using the Griess reagent, NED (0.1% in distilled water) and Sulphanilamide (1% in 5% H_3_PO_4_). DAF-2 DA was used to study intracellular NO as described elsewhere^56^ and observed under flow cytometer. Briefly, macrophages were scrapped and incubated in PBS containing DAF-2DA (7.0 μM) at 37 °C for 30 min and then assayed by BD FACS LSR Fortessa.

### Measurement of reactive oxygen species

To monitor the level of reactive oxygen species, the cell permeable probe DCFDA was used as described elsewhere^57^ and observed under Perkin-Elmer LS50B Spectrofluorometer or flow cytometer. Briefly, cells were treated with or without ALS of varying dose for indicated time, then scraped, washed and incubated in PBS containing DCFDA (20 mM) for 30 min at room temperature in the dark. Then fluorescence was measured using spectrofluorometer or flow cytometer.

### Confocal Microscopy

MΦs were harvested, fixed, and perforated and stained with primary and fluorochrome conjugated secondary antibody. After washing with TBS, slides were mounted using DAPI to visualize the nuclei in case of translocation of NF-κΒ or c-JUN. Specimens were mounted on glass slides.

During lysosome phagosome fusion assessment, CFSE was used to stain PLD before incubation and Lysotracker was used to locate acidic lysosome compartment. RAB5, RAB7, LAMP1, RAB9 and Cathepsin D were stained using primary and secondary antibodies. All specimens were observed under ANDOR Spinning disc confocal microscope with 60X or (60 × 1.6)X objective lens. The MFI for each image was calculated using Image J software. For co-localization analysis, Image J software produces various coefficients. Pearson’s correlation was used to analyze the association between green (CFSE) and red (Lysotracker red and PE for RAB5, RAB7, LAMP1 and RAB9) or blue (Cruzfluor 405 for Cathepsin D)^44^.

### Assessment of *in vitro* leishmanicidal activity

MΦs pre-treated with or without ALS were infected with CFSE labeled PLD, then fixed using 4% paraformaldehyde solution. To find viable parasites, MΦs infected with CFSE labeled PLD were detected by flowcytometry and percentage positive cell and MFI were deduced from the histogram^58^.

### Statistical analysis

Two-tailed t-test or one-way ANOVA followed by Bonferroni post hoc test was used for statistical analysis. P values < 0.05 were considered to be significant.

## Additional Information

**How to cite this article**: Banerjee, S. *et al.* Attenuated *Leishmania* induce pro-inflammatory mediators and influence leishmanicidal activity by p38 MAPK dependent phagosome maturation in *Leishmania donovani* co-infected macrophages. *Sci. Rep.* **6**, 22335; doi: 10.1038/srep22335 (2016).

## Supplementary Material

Supplementary Information
