# Supplementary material for: Attenuated Leishmania induce pro-inflammatory mediators and influence leishmanicidal activity by p38 MAPK dependent phagosome maturation in Leishmania donovani co-infected macrophages
Source: Sci Rep. 2016 Mar 1;6:22335. doi: 10.1038/srep22335 (PMC4772118; doi:10.1038/srep22335)
Supplement: Supplementary Information [file srep22335-s1.pdf]

# Supplementary Information

## Attenuated *Leishmania* induce pro-inflammatory mediators and influence leishmanicidal activity by p38 MAPK dependent phagosome maturation in *Leishmania donovani* co-infected macrophages

Somenath Banerjee[1], Dipayan Bose[1], Nabanita Chatterjee[1], Subhadip Das[1], Sreeparna Chakraborty[2], Tanya Das[2], and Krishna Das Saha[1]*

[1]Cancer Biology and Inflammatory Disorder Division, CSIR-Indian Institute of Chemical Biology, 4 Raja S. C. Mullick Road, Kolkata-700032, India.

[2]Department of Molecular Medicine, Bose Institute, Centenary Campus, P 1/12, C. I. T. Road, Scheme– VIIM, Kolkata – 700054, West Bengal, India.

* To whom Correspondence and request for reprints should be addressed:

Dr. (Mrs.) Krishna Das Saha

Cancer Biology and Inflammatory Disorder division, CSIR-Indian Institute of Chemical Biology

4, Raja S. C. Mullick Road, Kolkata-700032, India.

Telephone number: +91-9143258483

Fax number: +91 33 2473 5197, +91 33 2472 3967

E-mail address: krishnaiicb@yahoo.com

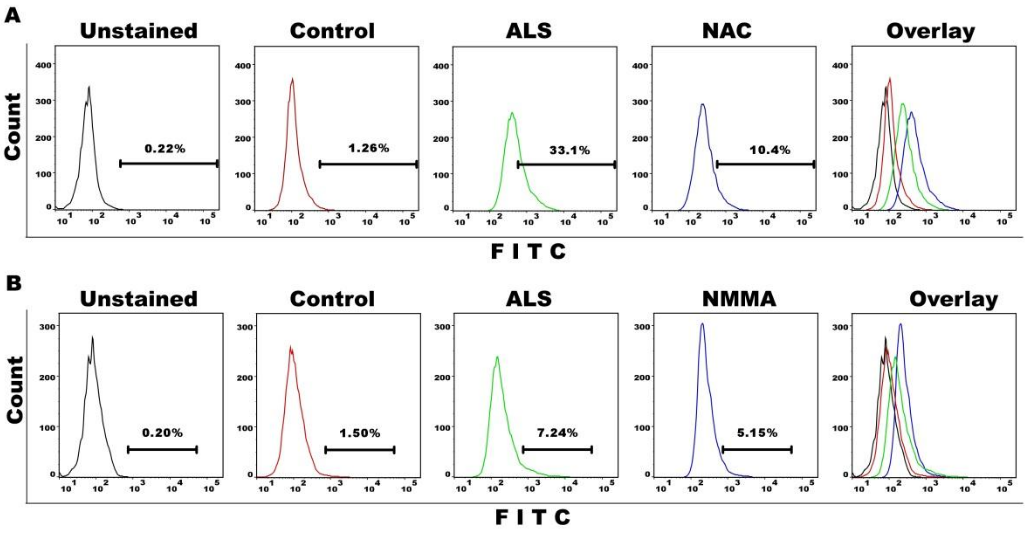


**Supplementary Figure S1. Down regulation of ROS and NO in ALS treated Mϕs by treatment of NAC and NMMA respectively.** MΦs were infected with $1\times10^7$ ALS for 4h, washed, and then treated with NAC (A) and NMMA (B). After 4h cells were washed and incubated for further 8h in fresh media. Then they are processed for ROS (A) and NO (B) detection using DCF-DA and DAF-FM respectively

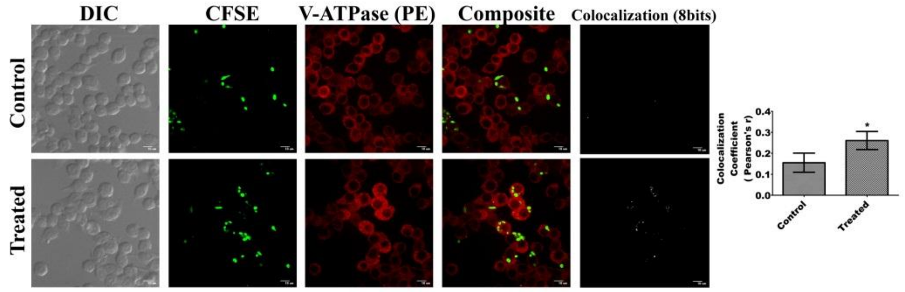


**Supplementary Figure S2. Co-localization of late phagosomal markers V-ATPase with CFSE labeled PLD.** RAW 264.7 cells were primed with $1\times10^7$ ALS for 4h, washed and fresh media was added. After 12h CFSE labeled PLD was added and incubated for further 12h. Co-localization of V-ATPase with CFSE labeled PLD was studied in MΦs. Colocalization index (Pearsons' r) were graphically represented (right panel). Data are representative as the mean ± SD and are the cumulative results from three independent experiments *p<0.05, **p<0.01, ***p<0.001.

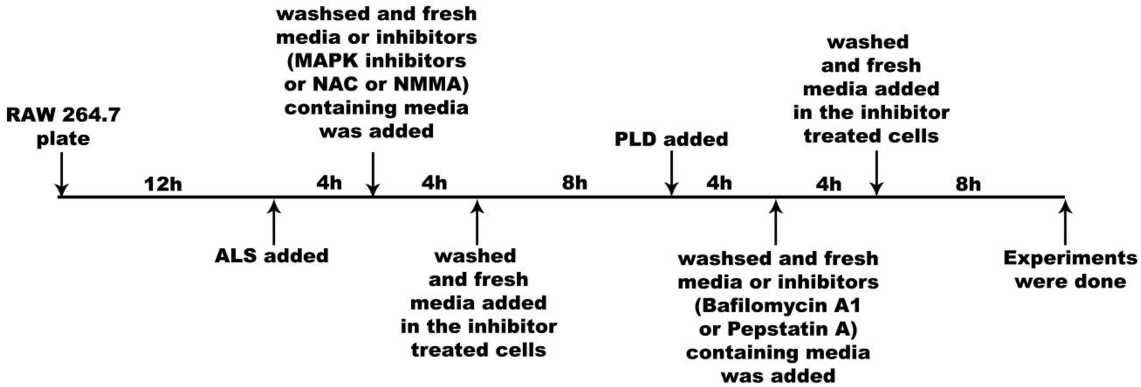


**Supplementary Figure S3. A schematic representation of the infection and treatment procedure followed throughout the experiment(s).**
